# The Coming Meeting at Galveston

**Published:** 1888-02

**Authors:** 


F. E. DANIEL, M. D., EDITOR AND PUBLISHER

THIS JOURNAL, by unanimous vote of the members, was made the Official Organ of the Austin District Medical Society.

COLLABORATORS:

*Dr. R. M. Swearingen., Austin.*
*Dr. B. E. Hadra, Austin.*
*Dr. Geo. Cupples, San Antonio.*
*Dr. T. C. Osborn, Cleburne, Texas.*
*Dr. E. J. Doering, Chicago.*
*Dr. Odo Betz, Germany.*
*Dr. J. W. McLaughlin, Austin.*
*Dr. T. J. Tyner, Austin.*
*Dr. J. F. Y. Paine, Galveston.*
*Dr. E. Goldmann, San Jose, Cal.*
*Dr. H. O. Marcy, Boston.*
*Dr. E. Meierhof, New York.*
*E. J. Deall, M. D., Fort Worth, Texas.*

### THE COMING MEETING AT GALVESTON.

Indications point to a large gathering. The season is propitious, the city and the occasion very attractive, and the able committee of arrangements have made ample provisions.

The President's chaste and eloquent appeal in behalf of social and scientific reunion, and a harmonious discussion of Medical Science, has gone out, broadcast over the State, and cannot fail to arouse an interest where none was felt before, or intensify that of the most zealous members. It is a stirring appeal; and the JOURNAL sincerely hopes, and cherishes the belief, that it may be, to the present agitated state of professional feeling—the ground-swell as it were, following the storm which stirred, and nearly wrecked, the Association at the last meeting—like a "peace, be still!" to the troubled waters.

It is to be hoped that members,—that *all true Physicians,*—will come to this meeting, feeling that it is a Convention of Medical Men, for the discussion of Medical Science and its advancement and elevation—the better interests of legitimate Medicine, and the wellfare of those entrusted to their care, and *not* a high court of errors and appeals; *not* a Star chamber, nor an inquisition; nor yet a court, whose sole business is to regulate the personal and professional relations and intercourse between individuals; *not* a chance nor opportunity to vent personal spite or ill will, nor a tribunal, like a police court, to entertain charges of a criminal nature.

The new Constitution and By-laws—proposed—clearly defines the duties of the Judicial Council. It seems to us that all differences between members of local or county societies, should—if brought before it—be referred back to such societies.

It is earnestly to be desired that the approaching meeting should be characterized by harmony and brotherly professional feeling; the *future welfare of the Organization depends upon it!* One more such meeting as the last would hopelessly wreck it; many members are almost disgusted, and while they are *devoted* to the Science of Medicine, humanitarians at heart, they say, better disband than meet to wrangle and stir up strife. We appeal to the *pride* of the members. If the Association can get once more into smooth water, its future is assured; we will have one of the grandest and most influential State organizations extant, potent for good; it will be, as in the recent past, pointed to as an example for emulation. Our transactions have challenged the admiration of the medical world for originality of research, and zeal in the cause; and if—in future—the volume shall go forth with less of scandal, and more of science, more of medical literature and less of litigation, we can still hope to bear the palm and lead the van. Come then, members, with hearts imbued with kindly feeling to each other, and fired by zeal in the good cause of medicine! Sink all jealousies and personal piques, leave at home, or throw away forever, all *prejudices*, and meet in the pride and prime of your professional manhood, and for the good of the cause and suffering humanity.

